# Combinations of multimodal neuroimaging biomarkers and cognitive test scores to identify patients with cognitive impairment

**DOI:** 10.3389/fnagi.2025.1650629

**Published:** 2025-08-13

**Authors:** Yuriko Nakaoku, Soshiro Ogata, Kiyotaka Nemoto, Chikage Kakuta, Eri Kiyoshige, Kanako Teramoto, Kiyomasa Nakatsuka, Gantsetseg Ganbaatar, Masafumi Ihara, Kunihiro Nishimura

**Affiliations:** ^1^Department of Preventive Medicine and Epidemiology, National Cerebral and Cardiovascular Center, Suita, Japan; ^2^Department of Psychiatry, Institute of Medicine, University of Tsukuba, Tsukuba, Japan; ^3^Department of Neurology, National Cerebral and Cardiovascular Center, Suita, Japan; ^4^Department of Biostatistics, National Cerebral and Cardiovascular Center, Suita, Japan

**Keywords:** MCI, cognitive impairment, neuroimaging biomarkers, community, MRI

## Abstract

**Background:**

Early detection of mild cognitive impairment (MCI), defined as the prodromal stage of dementia, is key to delaying the progression to dementia through lifestyle interventions and/or pharmacological treatments. This study aimed to develop and test new identification models for MCI in community settings based on multiple sources of clinical features, including neuroimaging biomarkers.

**Methods:**

This cross-sectional study analyzed cognitive testing and MRI examination data from 148 community-dwelling older adults in Nobeoka City. MCI was assessed using the Memory Performance Index from the MCI Screen. The variables used for model development were multisource features, including MRI-derived biomarkers and cognitive test scores. Finally, MCI identification models were developed using a penalized logistic regression model with an elastic net algorithm.

**Results:**

Among the 148 participants (mean age, 78.6 ± 5.2 years), 44.6% were identified as having MCI. The area under the curve for the elastic net model using baseline variables (i.e., age, sex, and education) and the multisource model were 0.74 (95% confidence interval, 0.59 to 0.89) and 0.81 (0.67 to 0.94) in the test datasets, respectively. The addition of neuroimaging biomarkers and cognitive test scores significantly improved the performance of the model identifying MCI (*p* = 0.012 by DeLong’s test). The structural, perfusion, and diffusion MRI-derived biomarkers remained in the identification model with variable selection with the elastic net algorithm, and were thus considered important variables.

**Conclusion:**

Our multisource elastic net model demonstrated high performance at detecting MCI, suggesting that the combination of multimodal neuroimaging biomarkers contributes to MCI discrimination.

## 1 Introduction

Early detection of mild cognitive impairment (MCI), a transitional state between normal aging and dementia, is key to delaying progression to dementia through multi-domain lifestyle interventions and/or pharmacological treatments (Wallin et al., 2016). In particular, the development and approval of disease-modifying therapies (DMTs) has led to a paradigm shift in Alzheimer’s disease (AD) therapy strategies in recent years (Cummings et al., 2024a). These advancements have increased the demand for simple and timesaving screening tests to detect cognitive impairment in community settings, and to facilitate subsequent referrals to AD specialists for a formal diagnosis and assessment of treatment eligibility. In general, MCI embraces a heterogeneous condition with complex neuropathological profiles, including neurodegenerative disease, cerebrovascular disease, or a mixture of different pathologies (Petersen et al., 2018). Until recently, most MCI studies focused on MCI due to AD. However, growing recognition of mixed-pathology cases among elderly dementia patients has expanded selection criteria in new data platforms, incorporating AD and related disorders (ADRD) (Mormino et al., 2025).

Despite the importance of early detection of cognitive impairment (i.e., MCI and dementia), a systematic review found that as high as 61.7% (95% confidence interval [CI]: 55.0% to 68.0%) of dementia cases in communities remain undetected (Lang et al., 2017). This suggests that there is no definitive screening system for people with cognitive impairment, and further suggests a lack of awareness of and access to cognitive screening. In Japan, the overall prevalence of MCI among individuals aged 65 and older was estimated at 15.5% (95% CI: 10.6% to 20.4%) in 2022, which translates to 5.59 million individuals (95% CI: 3.82 to 7.35 million) (Ninomiya, 2023). In the case of the AD continuum, initiating DMTs at an early AD stage (MCI to mild dementia) is hypothesized to provide greater clinical benefits than initiation after the onset of moderate dementia (Cummings et al., 2024b). Further, recent studies have highlighted the significant role of lifestyle interventions in preventing dementia (Kivipelto et al., 2020; Vidyanti et al., 2025). Given that most individuals with MCI reside in community settings, there is a pressing need for large-scale and rapid screening tools tailored to community dwellers.

Few studies have developed identification models using multimodal MRI features, including features from structural, diffusion, and perfusion MRI, for the detection of MCI or attempted to understand the cumulative effect of these features (Dang et al., 2023). Neuroimaging biomarkers are considered promising because they are non-invasive and objectively measurable. Previous studies have reported reductions in hippocampal and entorhinal cortex volumes in the brains of subjects with amnestic MCI compared to controls (Pennanen et al., 2004). More advanced MR techniques, such as diffusion tensor imaging (DTI) and arterial spin labeling (ASL), have provided insights into the integrity of white matter tracts and cerebral perfusion. Although these techniques have not yet been established in routine clinical use, they have been reported to have potential as biomarkers for detecting early cognitive decline or biomarkers for monitoring dementia progression (Dang et al., 2023). DTI is a promising biomarker because it can reveal changes in the microstructure of the white matter tracts selectively impaired in the early stage of ADRD (Lo Buono et al., 2020). Growing evidence further supports the utility of ASL in differentiating patients with AD from cognitively normal individuals (Kapasouri et al., 2022). A combination of multimodal MRI features would benefit complementarily from the structural, diffusion and perfusion viewpoints. This approach thus could provide a comprehensive identification system for MCI, to deepen our understanding of its onset and progression, and support clinicians in the timely detection and treatments of MCI.

Although neuropsychological testing is the standard diagnostic method for MCI, a previous study showed that a dementia prediction model combining multisource features (i.e., neuroimaging features, cognitive test scores and genetic factors), which provide complementary information, had the highest predictive ability (Payton et al., 2018).

Accordingly, the present study aimed to build a model for identifying MCI patients using multiple variables, including neuroimaging biomarkers and cognitive test markers, evaluate its performance, and assess the contribution of each biomarker in the best model to identify useful MCI biomarkers.

## 2 Materials and methods

### 2.1 Study population

This study utilized data from a cross-sectional telephone survey and a cross-sectional brain health check-up as a secondary analysis. The survey and check-up targeted older adults aged 71 to 95 years living in Nobeoka City, Miyazaki Prefecture, Japan. The survey was conducted between July 2021 and March 2023 as part of a public health service by Nobeoka City. Invitation letters were distributed to all eligible residents who met all of the following four criteria: (1) no moderate to severe cognitive impairment (corresponding to level II-A or higher on Independence in Daily Living of Elderly People with Dementia in the public long-term care insurance program), (2) no speech impairment, hearing impairment, or dysphonia, (3) no diagnosis of dementia, and (4) residing in their own homes (excluding those in long-term hospitalization or institutional care) within Nobeoka City. A total of 1,763 community-dwelling older adults participated in the telephone survey, of whom 151 applied for brain health check-ups.

The present study was conducted in accordance with the Declaration of Helsinki and Ethical Guidelines for Medical and Biological Research Involving Human Subjects. The research protocol was approved by the Ethics Committee of the National Cerebral and Cardiovascular Center (#R21064-3). Written informed consent of participants was not required because the data used in this study were de-identified prior to provision for analysis and remained anonymous at all stages, including data cleaning and statistical analysis.

### 2.2 MCI assessment

To assess cognitive function, the Japanese version of the MCI Screen (Cho et al., 2008), which is based on the 10-word immediate and delayed recall test of the Consortium to Establish a Registry for Alzheimer’s Disease (CERAD) battery (Morris et al., 1989), was administered. In this 10-min, computer-scored, staff-administered, cognitive test to screen for MCI and dementia stages of ADRD, a sophisticated scoring algorithm that analyzes the pattern of words recalled by each subject is used to calculate the Memory Performance Index (MPI) score according to the subject’s test results, age, education, and race. The validity and specificity of this test for differentiating between cognitively normal status (CN) and MCI have been described elsewhere (Cho et al., 2008), and accuracy, sensitivity, and specificity in discriminating MCI from CN have been reported to be 97% (95% CI, 97% to 98%), 94% (95% CI, 93% to 95%), and 89% (95% CI, 88% to 91%), respectively (Shankle et al., 2005). MPI scores of 49.8 and lower are classified as MCI and those greater than 49.8 as CN (Nogi et al., 2021).

### 2.3 Other cognitive tests and demographic data

Participants underwent cognitive tests at the hospital on the same day as the MRI scan. The tests included the Trail Making Test-B (TMT-B) (Reitan, 1958), the Logical memory II (LM) test of Wechsler Memory Scale-Revised (Wechsler, 1981) (Delayed paragraph recall, paragraph A only), and Verbal fluency test (VFT) (category “animal”) (Rosen, 1980). The LM test is widely used to assess verbal memory and considered a sensitive test for AD. Using a questionnaire, the following information was collected: age, sex, years of education, and past and current medical histories (hypertension, diabetes mellitus, hyperlipidemia, stroke, myocardial infarction, cancer, and chronic kidney disease).

### 2.4 MRI data acquisition

**FIGURE 1:**
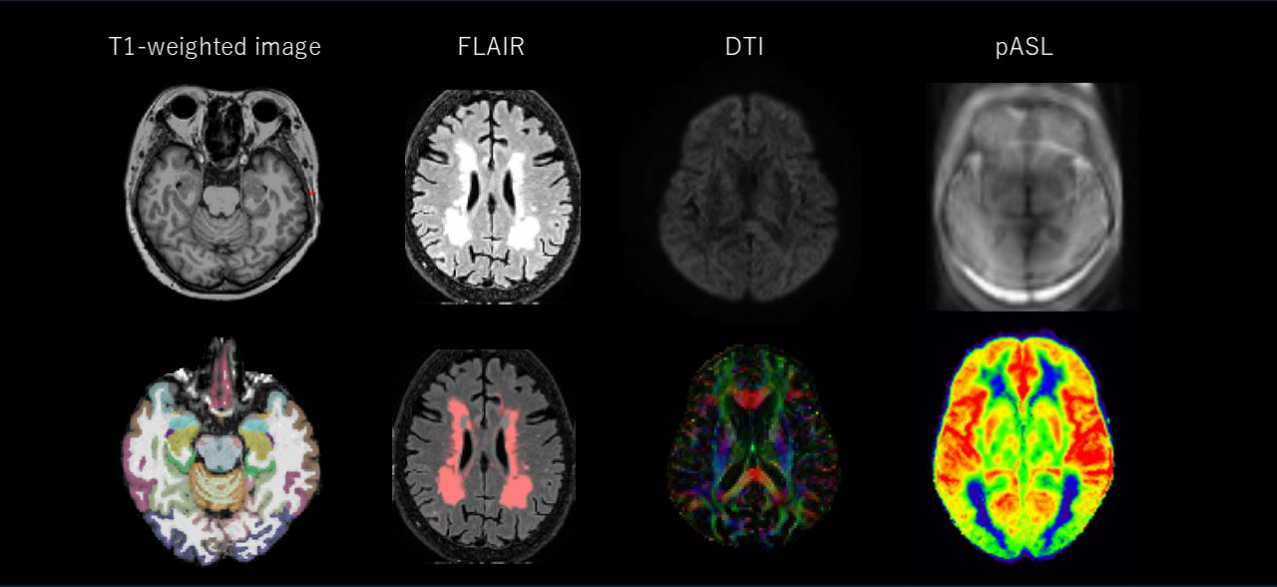
Representative MRI images. MRI images in the upper row are unprocessed images, while those in the lower row are processed images. The DTI color map shows the direction of the projection fibers (*z*-axis, blue), the association fibers (*y*-axis, green), and the subcortical fibers (*x*-axis, red). FLAIR, fluid-attenuated inversion recovery; DTI, diffusion tensor imaging; pASL, pulsed arterial spin labeling.

MRI scans were conducted on a 3T MAGMETOM Spectra MRI system (Siemens Healthineers, Erlangen, Germany) with the following sequences (Figure 1): (1) T1-weighted 3-dimensional (3D) imaging using a magnetization prepared rapid gradient echo sequence with repetition time (TR)/inversion time (TI)/echo time (TE) = 2,300/900/3.31 milliseconds (ms), field of view (FOV) = 240 mm × 256 mm, parallel imaging factor = 2, voxel size = 1 mm^3^ × 1 mm^3^ × 1 mm^3^, and flip angle = 8°; (2) T2-weighted 3D fluid-attenuated inversion recovery (T2-FLAIR) sequence with TR/TI/TE = 4,800/1,650/441 ms, FOV = 256 mm × 256 mm, parallel imaging factor = 2, voxel size = 1 mm^3^ × 1 mm^3^ × 1.2 mm^3^, 192 slices; (3) DTI using monopolar diffusion-weighted echo-planar imaging with 2 b values (0, 1,000 s/mm^2^) along 30 diffusion encoding directions, voxel size = 2 mm^3^ × 2 mm^3^ × 2 mm^3^, TR/TE = 11,500/101 ms, FOV = 232 mm × 232 mm; and (4) pulsed arterial spin labeling (pASL) using FAIR II labeling scheme, TR/TI/TE = 5,000/2,000/24.14 ms, FOV: 192 mm × 192 mm, voxel size: 3 mm^3^ × 3 mm^3^ × 4 mm^3^, bolus duration: 800 ms, flip angle: 180°, 8 dynamic scans, echo spacing = 0.84, turbo factor = 12, EPI factor = 21.

### 2.5 Image processing

FreeSurfer (Fischl, 2012) version 7.3.2 was used to obtain cortical thickness and volumetric measures from T1-weighted images. Briefly, this processing includes motion correction, removal of non-brain tissue, automated Talairach transformation, and intensity normalization for surface and intensity-based segmentation of the cortex, subcortical white matter, and deep gray matter volumetric structures (Fischl et al., 2002). Quality control (QC) on T1-weighted images and FreeSurfer segmentation through visual inspection were conducted, while the fsqc toolbox (Bedford et al., 2023) was applied to evaluate the signal-to-noise (SNR) values. The subjects were excluded if their SNR values were below the mean minus three standard deviations. Poor-quality images and images with identified brain tumors were also excluded. The “asegstats2table” and “aparcstats2table” commands were used to obtain the hippocampal volume, total intracranial volume (TIV) and cortical thickness values from each hemisphere. The mean hippocampal volume and cortical thickness of the entire cortex were calculated by averaging bilateral hippocampal volumes and cortical thicknesses.

ASL image processing was performed with ExploreASL (Mutsaerts et al., 2020), which is developed in MATLAB and includes the Computational Anatomy Toolbox (CAT12) (Gaser and Dahnke, 2024) for SPM 12 Statistical Parametric Mapping (SPM12) (Wellcome Centre for Human Neuroimaging, 2025) and the lesion segmentation toolbox (LST) (Schmidt, 2025) with the lesion prediction algorithm (LPA). ExploreASL included motion correction, quantification according to the ASL consensus paper (Alsop et al., 2015), rigid-body registration of the cerebral blood flow (CBF) map to a gray matter (GM) map from a segmented T1-weighted image, and spatial normalization to MNI space via the segmented T1-weighted image (Mutsaerts et al., 2020). As a parameter from CBF maps, GM-CBF was obtained. CBF reflects perfusion in mL blood/100 g tissue/min and was calculated in total GM regions of interest. Quantitative MRI analyses for WMH volume and GM-ICV ratio were also performed with ExploreASL.

Diffusion-weighted imaging was preprocessed using FMRIB Software Library (FSL) 6.0.4. First, eddy current distortion correction was performed using eddy_correct, followed by generation of fractional anisotropy (FA) images using dtifit. Tract-based Spatial Statistics (TBSS) was used to obtain a projection of all FA data onto a mean FA skeleton (Smith et al., 2006). Specifically, all FA images were first registered to the standard template (FMRIB58_FA_1 mm) using non-linear registration. Then, the mean FA image was created and thresholded (FA > 0.2) to create a mean FA skeleton. Finally, aligned FA of each subject was projected onto this skeleton, and the resulting data were fed into voxel-based cross-subject statistics. The same pipeline was used for mean diffusivity (MD) images, and registration warps produced for the subject FA image to FMRIB58_FA_1 mm were used to align these images to the same template. To avoid contamination of the skeleton by CSF partial volume effects, MD skeletons were masked using a standard FA skeleton thresholded at an FA value of 0.3. Finally, a histogram analysis was performed on the resulting MD skeletons, and the peak width of skeletonized mean diffusivity (PSMD) was calculated as the difference between the 95th and 5th percentiles of voxel-based MD values within the skeleton (Baykara et al., 2016). We also used histogram metrics of DTI-derived indices, specifically the median values of MD and FA.

### 2.6 Statistical analyses

Baseline characteristics are presented as median (interquartile range [IQR]) for continuous variables and number (%) for categorical variables. To develop identification models, the dataset was randomly split into a training dataset (70%) for developing identification models and a test dataset (30%) for assessing the prediction performance of the developed models. When developing identification models, continuous predictor variables were converted to z-scores by subtracting the mean values from the value of each variable and dividing by the standard deviation. Mean and standard deviation values were obtained from the training dataset.

First, to establish the predictive ability of the neuroimaging biomarkers (i.e., GM-ICV ratio, hippocampal volume, cortical thickness, TIV, WMH volume, gray matter CBF, FA median, MD median, and PSMD) for classifying cognitive status (MCI/CN), we assessed the area under the receiver operating characteristic (ROC) curve (AUC) of preliminary models in the training dataset.

Next, we developed three identification models to identify the cognitive status class (i.e., MCI or CN) in the training dataset. The baseline model included age, sex, and education. These variables were used because a previous study has developed a dementia prediction model using the same variables (C-statistic = 0.78; 95% CI: 0.76 to 0.81) (Machado-Fragua et al., 2021). In this study, the predictive model using age, sex, and education showed superior predictive performance compared with other models incorporating cognitive test scores. The structural imaging model included structural neuroimaging biomarkers (i.e., GM-ICV ratio, hippocampal volume, cortical thickness, and TIV) in addition to the variables included in the baseline model. The multisource model included neuroimaging biomarkers (i.e., structural, perfusion, and diffusion MRI-derived biomarkers) and cognitive test scores (TMT-B, LM test and VFT), in addition to variables included in the baseline model. The three identification models were developed by applying a penalized logistic regression model via an elastic net algorithm in the training dataset with 5-fold cross-validation to adjust parameters α and λ based on comparisons of model performance. This approach simplifies the selection of the most meaningful set of variables for predicting MCI status while minimizing dependency and redundancy by steering their coefficients toward zero. To account for multicollinearity, MD median values with a variable inclusion frequency (VIF) of 10 or more were excluded, because PSMD and MD median values are derived from the same MD variable.

Finally, two models (baseline model and multisource elastic net model with the most meaningful set of variables) were applied to the test dataset to evaluate their performance by the AUC. In addition, the accuracy, sensitivity, specificity, positive predictive value (PPV), and negative predictive value (NPV) were evaluated. AUCs of the baseline and multisource elastic net models were compared by DeLong’s tests.

We also examined the feature importance and calibration results in the multisource elastic net model. The feature importance of each variable was investigated to identify explainable features. Calibration plots were analyzed by dividing participants into quintiles to show the agreement between the predicted probability from the multisource elastic net model and the observed proportion of MCI, as recommended in the Transparent Reporting of a multivariable prediction model for Individual Prognosis or Diagnosis (TRIPOD) reporting guideline (Moons et al., 2015). The predicted probabilities and the observed proportions of MCI in each quartile were compared using the Hosmer–Lemeshow test (Hosmer et al., 1997) to test for the equality across all quartiles.

All statistical analyses were performed using the statistical software R (version 4.4.1) (R Core Team, 2024). All statistical significance tests were two-sided, using *p* < 0.05 as the level of statistical significance. Identification model development and model performance comparison were performed with the R “pROC” package (Robin et al., 2011) and the “caret” package (Kuhn, 2008).

## 3 Results

### 3.1 Clinical and radiological characteristics

In total, 148 of the 151 participants were included in the analysis after excluding 1 participant with large artifacts and 2 participants with a brain tumor. The median MPI score (IQR) was 52.6 (41.1, 64.8). We identified 66 (44.6%) participants as having MCI based on MPI scores ≤ 49.8. Median age of the entire study population was 78 (IQR, 74, 84) years, and there were 75 (50.7%) males. Demographic variables, cognitive test scores, and neuroimaging biomarkers in the training and test datasets are shown in Table 1.

**TABLE 1:** Clinical and radiological characteristics in training and test datasets.

	Training dataset (*n* = 103)	Test dataset (*n* = 45)
**Demographic variables**		
Age, years (median [IQR])	78 [75, 81]	78 [74, 84]
Male, *n* (%)	46 (45)	29 (64)
Education, years (median [IQR])	12 [9, 12]	12 [10, 13]
MCI, *n* (%)	43 (42)	23 (51)
**Comorbidity, *n* (%)**		
Hypertension	59 (59)	24 (56)
Diabetes mellitus	12 (12)	10 (24)
Hyperlipidemia	35 (36)	14 (33)
Stroke	9 (9)	4 (9)
Myocardial infarction	11 (11)	7 (17)
Cancer	19 (19)	7 (16)
Chronic kidney disease	1 (1)	2 (5)
**Neuroimaging biomarkers (median [IQR])**		
GM-ICV ratio	0.39 [0.37, 0.41]	0.37 [0.35, 0.39]
Hippocampal volume, mm^3^	7,166 [6,709, 7,682]	7,024 [6,720, 7,696]
Cortical thickness, mm	2.60 [2.54, 2.66]	2.59 [2.52, 2.62]
WMH volume, mm^3^	10 [5, 20]	13 [6, 22]
Gray matter CBF, ml/100 g/min	54 [40, 68]	53 [37, 68]
FA median	0.46 [0.44, 0.47]	0.45 [0.44, 0.47]
MD median, 10^–4^ mm^2^/s	7.48 [7.30, 7.66]	7.45 [7.33, 7.71]
PSMD, 10^–4^ mm^2^/s	3.62 [3.21, 4.04]	3.67 [3.42, 4.23]
TIV, cm^3^	1,494 [1,396, 1,612]	1,447 [1,369, 1,604]
**Cognitive test scores (median [IQR])**		
TMT-B	148 [98, 213]	145 [118, 209]
Verbal fluency test	15 [12, 17]	13 [11, 16]
Logical memory test	8 [5, 11]	7 [4, 11]

IQR, interquartile range; MCI, mild cognitive impairment; MRI, magnetic resonance imaging; GM, gray matter; ICV, intracranial volume; WMH, white matter hyperintensity; CBF, cerebral blood flow; FA, fractional anisotropy; MD, mean diffusivity; PSMD, peak width of skeletonized mean diffusivity; TIV, total intracranial volume; TMT-B, Trail Making Test-B. FA is a dimensionless index.

ROC curve analysis revealed AUCs in the range of 0.55–0.68 for neuroimaging biomarkers when each of them was used as an independent variable of a univariable model (Table 2). In particular, WMH volume (AUC, 0.68; 95% CI, 0.57 to 0.79) and PSMD (AUC, 0.69; 95% CI, 0.59 to 0.80) were suggested to be potentially useful (Table 2). However, no single neuroimaging biomarker achieved good model accuracy of AUCs > 0.7.

**TABLE 2:** Identification performance of neuroimaging biomarkers for MCI in the training dataset.

Neuroimaging biomarkers	AUC	95% confidence interval
GM-ICV ratio	0.63	(0.53–0.74)
Hippocampal volume	0.54	(0.43–0.66)
Cortical thickness	0.53	(0.41–0.64)
WMH volume	0.68	(0.57–0.79)
Gray matter CBF	0.59	(0.48–0.70)
FA median	0.62	(0.51–0.74)
MD median	0.61	(0.50–0.73)
PSMD	0.69	(0.59–0.80)
TIV	0.57	(0.46–0.69)

MCI, mild cognitive impairment; AUC, area under the curve; GM, gray matter; ICV, intracranial volume; WMH, white matter hyperintensity; CBF, cerebral blood flow; FA, fractional anisotropy; MD, mean diffusivity; PSMD, peak width of skeletonized mean diffusivity; TIV, total intracranial volume.

### 3.2 Development of the three elastic net models to identify MCI in the training dataset

**FIGURE 2:**
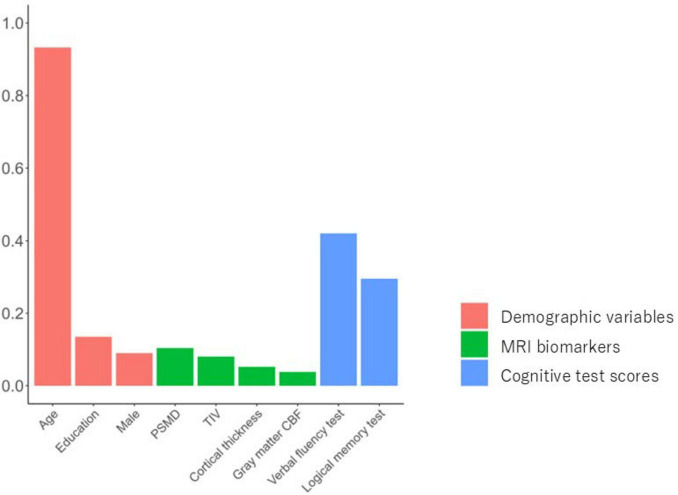
Nonzero coefficients for the multisource elastic net model. Bar plot shows the importance of features in the developed multisource elastic net model for MCI identification. PSMD, peak width of skeletonized mean diffusivity; TIV, total intracranial volume; CBF, cerebral blood flow.

A penalized logistic regression model selected all three demographic variables (age, sex, and education) for the baseline model. Six of the seven features (age, sex, education, cortical thickness, GM-ICV ratio and TIV) were selected in the structural imaging model; hippocampal volume was removed from the model because the coefficient was zero. Similarly, the final optimized multisource elastic net model used nine features: age, VFT, LM test, education, PSMD, sex, TIV, cortical thickness, and gray matter CBF, with coefficients of 0.93, 0.42, 0.30, 0.13, 0.10, 0.09, 0.08, 0.05, and 0.04, respectively. The coefficients of the hippocampal volume, GM-ICV ratio, WMH volume, FA median, and TMT-B were zero, and these were therefore removed from the multisource model (Figure 2).

### 3.3 Performance of elastic net models in the training and test datasets

**FIGURE 3:**
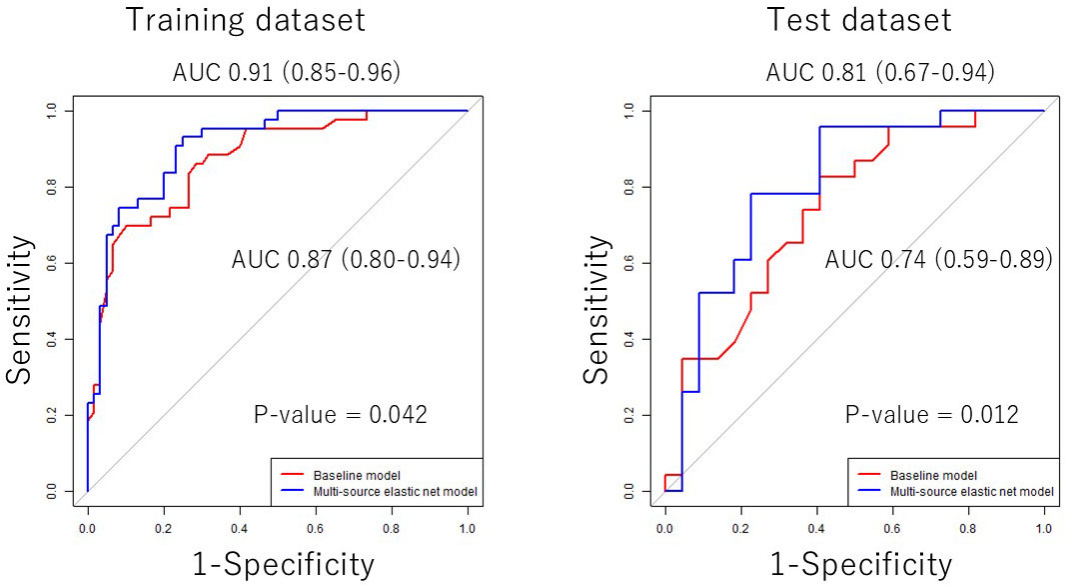
ROC curves and area under the curve (AUC) comparison of the baseline and multisource elastic net models for MCI identification in the training and test dataset. Statistical analysis was performed to compare AUCs of the baseline model and the multisource elastic net model (neuroimaging biomarkers and cognitive test scores as well as demographic variables). There were significant differences between the two models. The *P*-value is reported in each graph. AUC, area under the curve; ROC, receiver operating characteristic.

ROC curves of the baseline and multisource elastic net models in the training and test datasets are shown in Figure 3, and the performance of these models and the structural imaging model is shown in Table 3. In the training dataset, the AUC of the baseline model was 0.87 (95% CI: 0.80 to 0.94), the AUC of the structural imaging model was 0.90 (95% CI: 0.84 to 0.96), and the AUC of the multisource model was 0.92 (95% CI: 0.87 to 0.98). In the test dataset, the AUC of the baseline model was 0.74 (95% CI: 0.59 to 0.89), the AUC of the structural imaging model was 0.74 (95% CI: 0.60 to 0.89) and the AUC of the multisource model was 0.81 (95% CI: 0.67 to 0.94). The multisource elastic net model demonstrated better predictive accuracy than the baseline model (DeLong’s test, *p* = 0.012) (Figure 3).

**TABLE 3:** Identification performance of baseline, structural imaging and multisource elastic net models in training and test datasets.

Dataset	Training dataset			Test dataset		
Model	Baseline model	Structural imaging model	Multisource model	Baseline model	Structural imaging model	Multisource model
AUC	0.87 (0.80–0.94)	0.90 (0.84–0.96)	0.91 (0.85–0.96)	0.74 (0.59–0.89)	0.74 (0.60–0.89)	0.81 (0.67–0.94)
Accuracy	0.82 (0.73–0.89)	0.83 (0.74–0.89)	0.84 (0.75–0.90)	0.62 (0.47–0.76)	0.64 (0.49–0.78)	0.76 (0.60–0.87)
Sensitivity	0.65 (0.49–0.79)	0.72 (0.56–0.85)	0.74 (0.59–0.86)	0.52 (0.31–0.73)	0.61 (0.39–0.80)	0.74 (0.52–0.90)
Specificity	0.93 (0.84–0.98)	0.90 (0.79–0.96)	0.90 (0.79–0.96)	0.73 (0.50–0.89)	0.68 (0.45–0.86)	0.77 (0.55–0.92)
PPV	0.88 (0.71–0.96)	0.84 (0.68–0.94)	0.84 (0.69–0.94)	0.67 (0.41–0.87)	0.67 (0.43–0.85)	0.77 (0.55–0.92)
NPV	0.79 (0.68–0.88)	0.82 (0.70–0.90)	0.83 (0.72–0.91)	0.59 (0.39–0.78)	0.63 (0.41–0.81)	0.74 (0.52–0.90)

MCI, mild cognitive impairment; AUC, area under the curve; PPV, positive predictive value; NPV, negative predictive value. The baseline model included age, sex, and education as demographic variables. The multisource elastic net model included neuroimaging biomarkers and cognitive test scores (i.e., PSMD, total intracranial volume, cortical thickness, gray matter CBF, VFT, and LM test).

**FIGURE 4:**
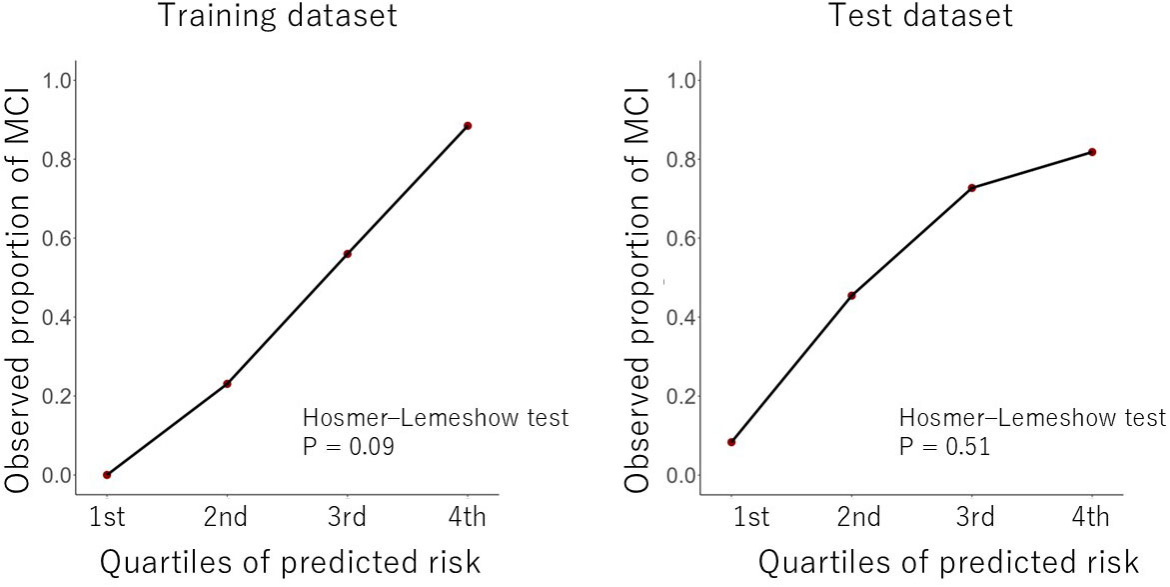
Calibration plots and Hosmer–Lemeshow test values for the developed multisource models in the training and test datasets The Hosmer–Lemeshow test indicated a good fit for the multisource elastic net model (*p*>0.05), suggesting that the model adequately fits the data.

Figure 4 shows calibration plots of observed proportion of MCI in each quartile of predicted probability using the multisource elastic net model. Both training and test models presented good calibration (Hosmer–Lemeshow test, *p* = 0.09 in the training dataset; *p* = 0.51 in the test dataset).

## 4 Discussion

In this community-based study of older adults, we developed a multisource elastic net model with high MCI discrimination accuracy combining demographic factors, cognitive test scores, and a various MRI-derived biomarkers. The addition of MRI-derived biomarkers and cognitive test scores to demographic variables (age, sex, and education) significantly improved MCI discrimination performance (AUC 0.81). While the strong contribution of demographic variables such as age, sex, and education in the multisource elastic net model is consistent with previous reports, this study newly shows that neuroimaging biomarkers are important variables, as they were retained in the model even after variable selection with the elastic net algorithm. In contrast, adding only structural MRI-derived biomarkers to the baseline model resulted in unchanged model performance (AUC 0.74). The observed contributions of variables from structural images, as well as those from diffusion and perfusion images, provide insights into the importance of combining complementary MRI-derived neuroimaging biomarkers.

### 4.1 High performance model for detecting MCI

Our finding that neuroimaging biomarkers contributed to the improved performance of our multisource elastic net model is in line with previous studies. Systematic reviews have identified more than 100 different dementia prediction models. However, some showed poor generalizability, particularly on external validation. Only six studies have developed dementia prediction models including MRI-derived variables, and their discriminative performance varied (AUC range: 0.55–0.92) (Brain et al., 2024). Furthermore, few studies have reported models which included MRI-derived biomarkers for MCI-specific outcomes. There are three possible reasons for this. First, MRI changes in people with MCI are subtle compared to those in dementia, making it difficult to achieve high identification accuracy. Second, MCI is not actively diagnosed because of the lack of therapies. Nevertheless, following the recent approval of DMTs for AD in 2023, the need to identify patients in the early stages of MCI has increased. Third, MRI scans for screening MCI are relatively expensive, requiring approximately 30 min. Nevertheless, Japan has a considerable number of MRI machines (approximately 7,500; 59.8 per 1 million people) (Organisation for Economic Co-operation and Development, 2023) and a brain health check-up system called brain docks. Consequently, many healthy people in Japan undergo brain MRI screening.

Models for detecting MCI are rare. Moreover, only one study has reported the use of MRI-derived biomarkers as variables. A previous study in a community-based setting reported an MRI-based MCI identification model with an AUC of 0.61 (0.58–0.64) (Bouts et al., 2019). Herein, the inclusion of many variables including age, sex, education, and cognitive test scores, as well as MRI-derived biomarkers, likely improved the accuracy of our model.

### 4.2 Neuroimaging biomarkers relevant for detecting MCI

After feature selection using the elastic net algorithm, four neuroimaging biomarkers (PSMD, TIV, cortical thickness, and gray matter CBF) were retained in the multisource elastic net model, indicating utility in detecting MCI. This aligns with previous research showing that structural MRI features are closely associated with MCI (Forouzannezhad et al., 2020). However, single modality biomarkers often lack sufficient accuracy for early diagnosis. Therefore, biomarker combinations are recommended (Lombardi et al., 2020). However, which neuroimaging biomarkers should be used, in what combinations, and which brain functions they reflect currently remain unclear. The present study suggests that white matter biomarkers (DTI-derived biomarkers [e.g., PSMD and FA] and WMH) may be associated with both memory and executive function (Supplementary Table 1). Biomarkers of atrophy, such as hippocampal volume, may be associated with memory function (Supplementary Table 2). These findings provide an opportunity for future studies to elucidate the functional aspects of neuroimaging biomarkers.

### 4.3 PSMD is a selected neuroimaging biomarker of MCI

The present study showed that PSMD was the most important neuroimaging biomarker for identifying MCI in our multisource elastic net model. Notably, this is the first study to report the incorporation of PSMD from DTI into an MCI identification model. An increasing number of studies have demonstrated that progressive WM degeneration and demyelination are important pathological characteristics of ADRD (Nasrabady et al., 2018). PSMD is a novel imaging marker for small vessel disease (SVD) based on skeletonization and histogram analysis of diffusion MRI data (Baykara et al., 2016), and is associated with processing speed, memory, and general cognitive ability (Deary et al., 2019). In the present study, PSMD was associated with all cognitive test scores (VFT, LM test, TMT-B, and MPI score) across various subdomains (memory, language, and executive function) (Supplementary Table 1). Moreover, a more recent study reported that PSMD is not only a marker of cerebral small vessel disease, as PSMD values are also significantly higher in patients with ADRD (Luo et al., 2023). This is potentially because WM plays a role in information transmission and communication within the brain network. Overall, these findings indicate that PSMD derived from DTI images may be a promising biomarker for early MCI detection.

### 4.4 CBF is a selected neuroimaging biomarker of MCI

In the present study, gray matter CBF was a good contributor to MCI detection in the multisource elastic net model. A previous systematic review and meta-analysis of 244 studies with 13,644 participants further concluded that significant decreases in CBF from the precuneus to the posterior cingulate and from the temporal-parietal regions to broader areas accompany the progression from healthy controls to MCI and ADRD (Zhang et al., 2021), thus supporting the inclusion of CBF in the AD research framework. Although ASL perfusion imaging is not routinely performed in clinical practice in ADRD, CBF has long been studied as a regional marker of brain function and is increasingly being studied in research (Alsop et al., 2010). One reason for this is that vascular dysregulation derived from ASL images is the earliest pathological event in the progression of the ADRD continuum (Iturria-Medina et al., 2016). ASL MRI is a non-invasive technique for quantifying CBF without the use of exogenous tracers (Alsop et al., 2015) and can be repeated many times across progression through the ADRD continuum.

### 4.5 Implementation of the developed model

The multisource elastic net model developed in this study can be used in a brain dock, a preventive system to check brain health unique to Japan (Morita, 2019) which includes MRI and cognitive function tests. Although DTI and ASL images are not yet standard in brain docks, they can be readily added by setting up imaging sequences and extending the imaging time by several minutes. This would allow the assessment of white matter microstructural integrity and brain function, in addition to structure. Multicenter brain imaging databases in Europe now include DTI and ASL images as advanced MRI sequences. Given the limited availability of PET scans and genetic data, these were not included in the model developed in this study. Our model could help identify individuals at a high risk of MCI in the general population and provide an opportunity to recommend hospital visits, allowing for the possibility of early intervention and delayed dementia.

### 4.6 Strengths and limitations

The primary strength of this study is that the developed model can be applied to MCI screening in the community. By assessing multiple brain aspects, including sequences other than structural imaging, the model allows for the identification of patients with MCI who have not reached the hospital and/or are underdiagnosed. Recent studies have further reported that the early detection of cognitive decline (i.e., MCI) and implementation of multimodal lifestyle-based interventions could prevent progression to dementia (Kivipelto et al., 2020). Further, we identified MRI-derived biomarkers that could be used in MCI prediction models. It is now widely recognized that biomarker-based stratification is necessary to optimize the content of these interventions. Biomarkers derived from multiple brain MRI sequences can complement each other and could potentially aid in the categorization of MCI (Marquez and Yassa, 2019). Therefore, further randomized controlled trials (RCTs) are warranted. These could help categorize patients with MCI and hold promises for personalized medicine.

This study has several limitations. First, the sample size was limited because MRI scans conducted by Nobeoka City as part of municipal projects were used as the dataset. Second, the subjects identified as having MCI in this study could not be categorized as having MCI due to AD because their amyloid pathology was not assessed. Third, because this study utilized survey data collected by Nobeoka City as a secondary analysis, information on MCI subtypes was not included and was therefore unknown. However, the model developed in this study was aimed to detect those at a high risk of cognitive decline, as it is intended to serve as a screening tool in the community. Further studies using a cohort of patients with a confirmed diagnosis, including those diagnosed with MCI subtypes, are required. We believe that neuroimaging biomarkers, which reflect different brain functions depending on the brain region, can be utilized to develop prediction models for MCI subtypes and prognosis.

## 5 Conclusion

We developed a multisource elastic net model to detect MCI within a community-dwelling cohort using demographic variables, cognitive test scores, and multimodal neuroimaging biomarkers, and validated its performance (AUC = 0.81). Our findings suggest that biomarkers derived from MRI, including diffusion and perfusion images, may contribute to the diagnosis of MCI. Multimodal MRI may play an important role in objectively assessing mild signs of cognitive decline, paving the way for the more accurate and efficient detection of individuals in the early ADRD continuum.

## Data Availability

The data analyzed in this study is subject to the following licenses/restrictions: This study utilized data from a cross-sectional telephone survey and a cross-sectional brain health check-up as a secondary analysis. The survey and check-up targeted older adults aged 71 to 95 years living in Nobeoka City, Miyazaki Prefecture, Japan. The survey was conducted between July 2021 and March 2023 as part of a public health service by Nobeoka City. Requests to access these datasets should be directed to KN, knishimu@ncvc.go.jp.
