# Supplementary material for: Combinations of multimodal neuroimaging biomarkers and cognitive test scores to identify patients with cognitive impairment
Source: Front Aging Neurosci. 2025 Aug 13;17:1650629. doi: 10.3389/fnagi.2025.1650629 (PMC12380820; doi:10.3389/fnagi.2025.1650629)
Supplement: Supplementary file 1 [file Table_1.docx]

Supplementary Material

# Association of neuroimaging biomarkers with cognitive test scores

For each cognitive test score assessing different domains of cognitive function (e.g., memory and executive function), generalized linear models with normal distribution were used to evaluate the association with neuroimaging biomarkers, adjusted for covariates (i.e., age, sex, and education) in the combined training and test datasets.

The generalized linear models indicated that higher PSMD was associated with worse cognitive test scores. Moreover, in terms of the subdomains of cognitive function, PSMD was associated not only with memory but also with executive function and language domains. (MPI score [adjusted β = -1.99; 95% CI, -3.99 to 0.01; p = 0.05], TMT-B [adjusted β = 21.14; 95% CI, 7.35 to 34.93; p = 0.003], VFT [adjusted β = -0.75; 95% CI, -1.39 to -0.12; p = 0.02], and LM test [adjusted β = -0.95; 95% CI, -1.58 to -0.32; p = 0.004]) (Supplementary Table S1). FA median values derived from DTI images were associated with TMT-B and LM test. WMH was a predictor of MPI score, TMT-B, and LM test (Supplementary Table S1). Biomarkers of white matter of the brain (DTI image-related biomarkers [e.g., PSMD and FA] and WMH) were suggested to be associated not only with the memory domain but also the executive function domain. GM-ICV ratio was a predictor of MPI score and LM test. Hippocampal volume was associated with LM test (Supplementary Table S2). Biomarkers of brain atrophy (GM-ICV ratio and hippocampal volume) showed that brain atrophy was associated with subdomains of memory.

# Supplementary Tables

## Supplementary Table1. Adjusted coefficients for associations between cognitive test markers and neuroimaging biomarkers of white matter of the brain

| Outcome | PSMD | | FA median | | WMH volume | |
| --- | --- | --- | --- | --- | --- | --- |
|  | Beta  (95% confidence interval) | P-value | Beta  (95% confidence interval) | P-value | Beta  (95% confidence interval) | P-value |
| MPI score (memory domain) | -1.99  (-3.99 to 0.01) | 0.05 | -0.17  (-2.25 to 1.91) | 0.87 | -2.26  (-4.28 to -0.23) | 0.03 |
| Logical memory test of WMS-R (memory domain) | -0.95  (-1.58 to -0.32) | 0.004 | 0.86  (0.21 to 1.51) | 0.01 | -0.89  (-1.53 to -0.25) | 0.01 |
| TMT-B (attention, executive function) | 21.14  (7.35 to 34.93) | 0.003 | -23.15  (-36.60 to -9.70) | <0.001 | 16.31  (2.48 to 30.13) | 0.02 |
| Verbal fluency test (language domain) | -0.75  (-1.39 to -0.12) | 0.02 | 0.35  (-0.31 to 1.01) | 0.3 | -0.51  (-1.16 to 0.14) | 0.12 |

Abbreviations: WMH, white matter hyperintensity; FA, fractional anisotropy; PSMD, peak width of skeletonized mean diffusivity; MPI, memory performance index; TMT-B, Trail Making Test-B; WMS-R, Wechsler Memory Scale-Revised.

## Supplementary Table2. Adjusted coefficients for associations between cognitive test markers and neuroimaging biomarkers related to brain atrophy

| Outcome | GM-ICV ratio | | Hippocampal volume | | Cortical thickness | |
| --- | --- | --- | --- | --- | --- | --- |
|  | Beta  (95% confidence interval) | P-value | Beta  (95% confidence interval) | P-value | Beta  (95% confidence interval) | P-value |
| MPI score (memory domain) | 2.37  (0.23 to 4.52) | 0.03 | 0.71  (-1.38 to 2.80) | 0.51 | -0.02  (-2.00 to 1.95) | 0.98 |
| Logical memory test of WMS-R (memory domain) | 0.98  (0.30 to 1.65) | 0.01 | 0.88  (0.23 to 1.53) | 0.01 | 0.33  (-0.29 to 0.96) | 0.3 |
| TMT-B (attention, executive function) | -10.12  (-24.76 to 4.52) | 0.18 | -9.43  (-23.78 to 4.92) | 0.2 | 1.8  (-11.59 to 15.19) | 0.79 |
| Verbal fluency test (language domain) | 0.58  (-0.10 to 1.27) | 0.1 | -0.18  (-0.85 to 0.48) | 0.59 | -0.06  (-0.68 to 0.57) | 0.86 |

Abbreviations: GM, gray matter; ICV, intracranial volume; MPI, memory performance index; TMT-B, Trail Making Test-B; WMS-R, Wechsler Memory Scale-Revised.
